# Blunt chest wall trauma as a precipitant of late peri-implant haematoma and implant rupture: a case report

**DOI:** 10.1093/jscr/rjag411

**Published:** 2026-05-31

**Authors:** Krzysztof Sosnowski, Chloe Jordan, Charles M Malata

**Affiliations:** School of Clinical Medicine, University of Cambridge, Hills Rd, Cambridge CB20SP, United Kingdom; Department of Plastic and Reconstructive Surgery, Addenbrooke's Hospital, Cambridge University Hospitals NHS Foundation Trust, Hills Rd, Cambridge CB2 0QQ, United Kingdom; Department of Plastic and Reconstructive Surgery, Addenbrooke's Hospital, Cambridge University Hospitals NHS Foundation Trust, Hills Rd, Cambridge CB2 0QQ, United Kingdom; Cambridge Breast Unit, Addenbrooke's Hospital, Cambridge University Hospitals NHS Foundation Trust, Hills Rd, Cambridge CB2 0QQ, United Kingdom; Anglia Ruskin University School of Medicine, Anglia Ruskin University, East Rd, Cambridge CB1 1PT, United Kingdom

**Keywords:** breast augmentation, blunt chest trauma, implant rupture, peri-implant haematoma, capsular contracture, silicone implants, augmentation mammaplasty, mastopexy, implant complications

## Abstract

Whilst perioperative complications of implant-based breast surgery are well described in literature, late complications are comparatively uncommon, including anaplastic large cell lymphoma, late seroma, and late haematoma. The latter is rarely reported, and its aetiology remains a subject of ongoing debate with proposed mechanisms including direct trauma and capsular microfracture. We present a case in which low velocity blunt chest wall trauma precipitated concurrent implant rupture and late peri-implant haematoma 17 years following cosmetic breast augmentation (outside the perioperative period), in a patient with severe capsular contracture, requiring complex single-stage revisional surgery. This case contributes to the limited body of evidence on late postoperative complications of cosmetic breast augmentation in particular and implant-based breast surgery in general and highlights the diagnostic challenges inherent to the ‘silent rupture’ phenomenon. Clinicians should maintain a low threshold for implant assessment following blunt chest trauma, regardless of the velocity of the inciting injury.

## Introduction

Breast augmentation with implants is a common aesthetic surgical procedure, being the third commonest cosmetic operation performed globally [1]. Long-term complications of breast implant surgery include capsular contracture (CC), late peri-prosthetic seroma, implant rupture, and the rare breast implant-associated anaplastic large cell lymphoma (BIA-ALCL) [2]. As opposed to perioperative complications, which are well described in the literature, late complications are comparatively uncommon.

Whilst haematoma following breast implants occurs with a frequency of ~1% in the early postoperative period, late hematomas (>6 months postoperatively) remain poorly described, with ~20 published cases reported in the literature [3, 4]. As a result, its aetiology is poorly understood, with direct trauma, capsular microfracture, and chronic periprosthetic inflammation proposed as contributing mechanisms [5].

Similar to haematomas, implant rupture most commonly occurs intraoperatively due to instrument perforation or unrecognized implant defects, whereas postoperative rupture outside this context is uncommon [6]. What’s rarer is a combination of an implant rupture and a significant hematoma caused by direct blunt trauma to the chest. We therefore report such a case occurring in a 69-year-old who sustained low velocity trauma, causing a fractured humerus, swelling of the breast from the collection of blood and severe CC or exacerbation of CC.

## Case presentation

A 69-year-old female with a background of hypothyroidism and fibromyalgia underwent bilateral breast augmentation with round moderate plus silicone gel breast implants in January 2008. She re-presented in June 2025 following a mammogram in the preceding months that identified a ruptured right breast implant. The patient attributed this to a mechanical fall 18 months prior, where she landed onto the right side of her chest and sustained associated bruising. The fall was further complicated by a humeral fracture managed conservatively and persistent right breast pain.

**Figure 1:**
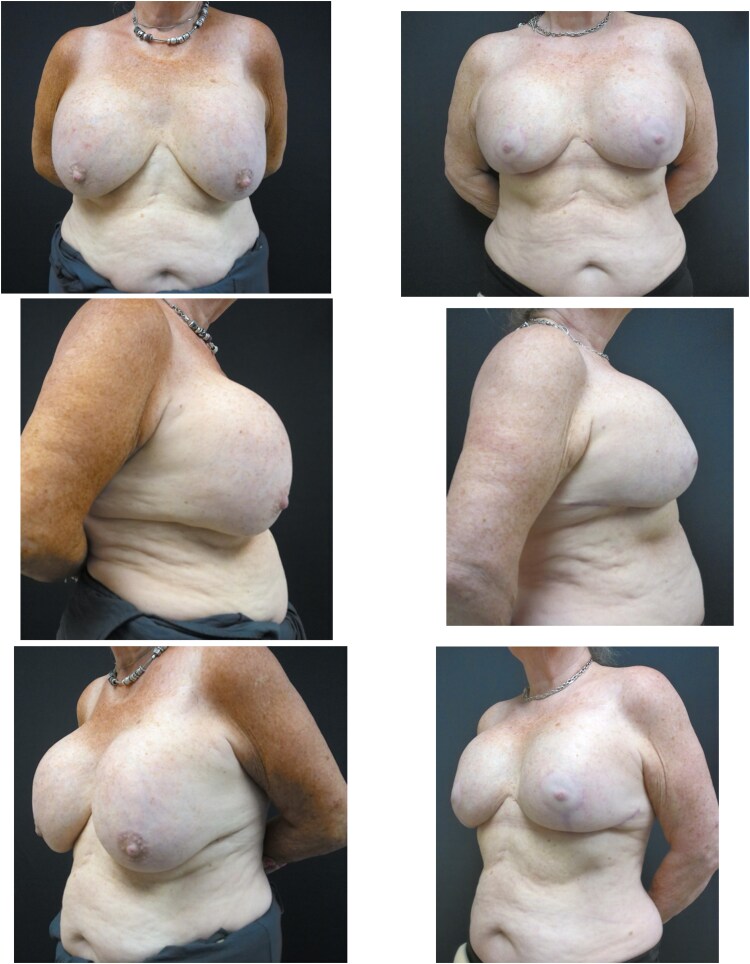
Pre-operative photos shown in the left sided column showing bilaterally augmented and asymmetrical breasts with grade 2 ptosis and 4 capsular contracture. There is evidence of excessive superior pole fullness due to severely encapsulated subglandular implants most pronounced on the right index side. Post-operative photos (right sided column) following bilateral total en bloc capsulectomies and implant exchange to smaller Mentor™ CPG™ textured-surface cohesive III silicone implants placed into neo-subpectoral pockets with concomitant T-scar mastopexy and dermoglandular sling auto. There is improved symmetry and shape of the now smaller breasts.

Clinical examination revealed enlarged breasts with excessive superior pole fullness due to severely encapsulated subglandular implants, accentuating a marked bilateral ‘waterfall’ deformity, most pronounced on the right index side (Fig. 1). Breast ptosis was graded as Regnault grade:2 bilaterally, with nipple position approaching grade:3, alongside marked overall breast asymmetry (Fig. 1). Capsular contracture of the index right breast was Baker grade:4 with associated distortion of the right inframammary fold.

Surgical planning was undertaken in accordance with the patient’s wishes for only a modest reduction in breast size whilst maintaining fullness of her breasts, despite the offer of explantation without re-augmentation, but with tissue rearrangement in the form of a mastopexy. Intraoperative findings demonstrated a large, liquefied peri-implant haematoma of the right breast and severe bilateral capsular contractures (Video 1). Silicone extravasation was confirmed bilaterally from ruptured silicone gel implants, with more extensive involvement on the right.

**Figure 2:**
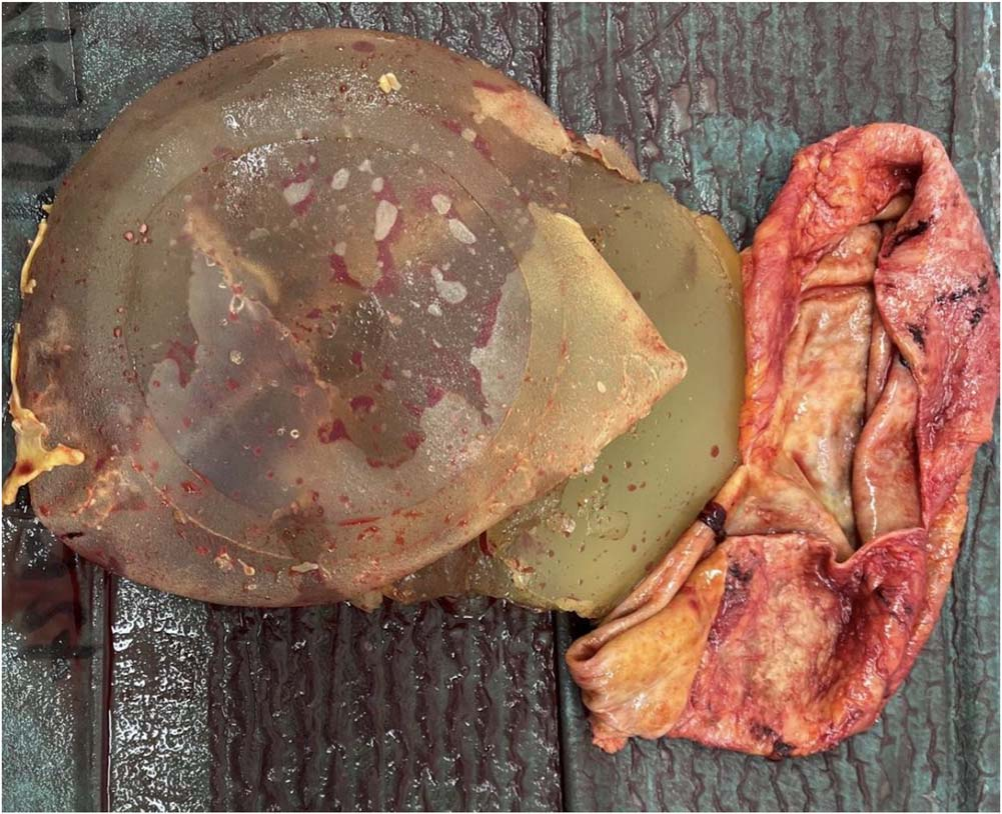
Ruptured right breast implant with capsule. The tell-tale features of the peri-implant haematoma are visible.

During revision, smaller Mentor™ textured silicone implants were placed into a neo-subpectoral plane with concomitant superomedial pedicle T-scar mastopexy and auto-augmentation using dermo-parenchymal slings suspended to the pectoralis major muscle, thus providing additional support of the implants. Bilateral total *en bloc* capsulectomies were performed (Fig. 2). Histological analysis of the capsular tissue demonstrated scar tissue, foreign body reaction and lymphoid infiltrate, necessitating immunohistochemical staining, which excluded BIA-ALCL.

Despite minor T-junction healing problems and a skin reaction to the dressings in the early postoperative period, by 5 months the patient had achieved an aesthetically acceptable outcome with uplifted, smaller and more symmetrical breasts consistent with her preoperative goals of mildly reduced reconstructed breast volume.

## Discussion

The patient herein reported presented 18 months following the traumatic event, after mammographic screening identified implant rupture. This delayed presentation is consistent with the phenomenon of ‘silent rupture’, which is inherent to silicone implants and is characterized by an absence of severe symptoms in the initial phase, thereby delaying diagnosis [6]. When implant rupture remains confined to the periprosthetic capsule, patients may remain asymptomatic or report mild tightness, breast distortion, or discomfort [7]. In the context of low-velocity traumatic incidents such as falls, delayed diagnosis is more likely, as patients and clinicians may underestimate the potential for implant injury.

A peri-implant haematoma was suspected preoperatively based on the patient’s report of immediate bruising following the fall and was subsequently confirmed intraoperatively. Haematoma is a recognized complication of breast implant surgery, typically occurring within the first 3 days of the perioperative period. Its occurrence months to years later is rare, with ˂20 cases documented in the literature [4]. Late haematoma characteristically presents as progressive breast swelling or a mass-like deformity, with diagnostic workup typically including computed tomography and magnetic resonance imaging [8].

Additionally, the patient had developed significant capsular contracture, which is a recognized long-term complication of implant-based surgery, with increasing incidence over time, having undergone primary augmentation 17 years prior [9]. The occurrence of late peri-implant haematoma is significantly associated with the development of capsular contracture [10]. Proposed mechanisms for these include capsular microfracture, erosion of capsular vessels, traction injury to such vessels, capsule-implant interface friction from CC, chronic inflammation [4]. In this case, it is difficult to determine whether the capsular contracture was pre-existing or was caused by or exacerbated by hematoma. Early postoperative haematoma is a well-known aetiology/cause of capsular contracture [11]. The combination of severe capsular contracture, bilateral implant rupture, breast ptosis, and asymmetry necessitated simultaneous management of bilateral total capsulectomy, haematoma evacuation, implant exchange, and mastopexy within a single operative encounter, which is seldom described in the literature, particularly at this interval following primary augmentation. Despite the complexity of the intraoperative findings, a satisfactory aesthetic outcome was achieved (Fig. 2).

## Conclusion

This case demonstrates that blunt chest wall trauma may precipitate concurrent implant rupture and late peri-implant haematoma outside the perioperative period, with simultaneous progression of capsular contracture in a patient with long-standing implants. Each of the first two findings is rare in isolation, and their co-occurrence following a single traumatic event managed with single-stage surgical revision and mastopexy has, to our knowledge, not been previously described. Furthermore, this case illustrates the surgical complexity encountered during late revisional breast surgery, requiring simultaneous management of multiple pathologies.

Clinicians should maintain a low threshold for implant assessment following blunt chest trauma, regardless of the velocity of the inciting injury to mitigate the diagnostic delays inherent to the silent rupture phenomenon.

## Supplementary Material

VIDEO-2026-04-14-15-41-43_rjag411 Ruptured right implant with associated haematoma.
